# Simultaneous Occurrence of Well-Differentiated Papillary Mesothelial Tumor and Multicystic Mesothelioma

**DOI:** 10.3390/diagnostics16142211

**Published:** 2026-07-15

**Authors:** Dražen Miličić, Snježana Tomić, Boris Delić, Marko-Dražen Mimica

**Affiliations:** 1 Department of Gynaecology and Obstetrics, University Hospital of Split, 21000 Split, Croatia; drazen.milicic@kbsplit.hr (D.M.); boris.delic@kbsplit.hr (B.D.); 2 Department of Pathology, Judicial Medicine, and Cytology, Division of Pathology, University Hospital of Split, 21000 Split, Croatia; stomic@mefst.hr; 3 School of Medicine, University of Split, 21000 Split, Croatia; 4 Faculty of Health Sciences, University of Split, 21000 Split, Croatia

**Keywords:** well-differentiated papillary mesothelial tumor (WDPMT), multicystic mesothelioma, adnexal tumor

## Abstract

We report a rare case of simultaneous well-differentiated papillary mesothelial tumor (WDPMT) and multicystic mesothelioma. The concurrent occurrence of these entities is exceptionally uncommon and may pose challenges during surgical intervention. Both lesions are rare mesothelial proliferations that are often identified incidentally during surgery. Well differentiated papillary mesothelial tumor (WDPMT) is a neoplastic mesothelial proliferation of low malignant potential, which predominantly involves the peritoneum of young women. Microscopically it is composed of branching plump papillae lined by a single layer of bland cuboidal mesothelial cells. Cytologic atypia is minimal, and mitoses usually absent (or at most 1 mitosis per 10 high power fields). Differential diagnosis includes: 1. diffuse malignant peritoneal mesothelioma, 2. localized malignant peritoneal mesothelioma, 3. serous borderline tumors. A 41-year-old patient was admitted for laparoscopic surgery due to a tumor mass localized near the left ovary. Ultrasound examination revealed solid lesion adjacent to the left ovary, exhibiting central vascularization. The patient was diagnosed with the solid tumor mass during routine gynecological examination. The mass was extraovarian and positioned between the left ovary and the uterus. Over the course of the next 18 months, the tumor increased 5 cm in size. Given the fact that the patient presented with no symptoms, such as abdominal pain, distension or ascites, and that the tumor markers were not elevated, additional radiological imaging such as CT or MRI of the abdomen and pelvis was not performed. Intraoperatively, the mass appeared as an extraovarian solid tumor of soft consistency positioned between the left ovary and the uterus. Extirpation of the tumor was performed. Frozen section analysis of the extirpated tumor mass confirmed the diagnosis of WDPMT. During the laparoscopic procedure, a left adnexectomy was also performed and multiple biopsies of white nodules were found dispersed in the peritoneal cavity. Final patohystological analysis concluded that these nodules were peritoneal inclusion cysts. Almost three years after the procedure, the patient is without symptoms and regular clinical and ultrasound examinations were performed every six months, which showed no signs of recurrence or dissemination of the primary tumor. Recognizing well-differentiated papillary mesothelial tumor and multicystic mesothelioma is critical for accurate differential diagnosis from malignant mesothelioma. Awareness of these entities informs appropriate surgical management and follow-up protocols.

**Figure 1:**
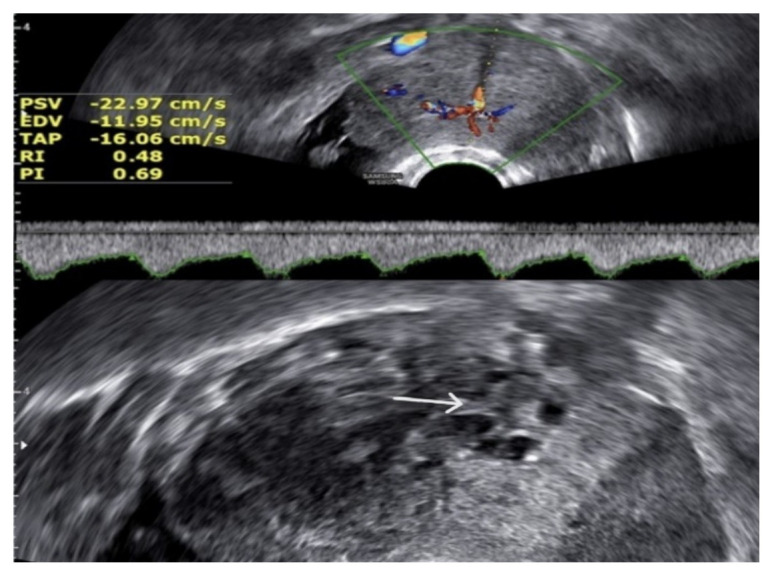
A 41-year-old gravida 1, para 1, female patient was admitted to our department for a laparoscopy because of a solid tumor mass positioned near the left ovary (ovary marked with arrow), diagnosed 18 months before admission during routine gynecological examination. Regular transvaginal ultrasound examinations were performed, which showed a slow but steady increase in the size of the solid tumor mass. From the fist to the last ultrasound examination, it increased in diameter from 3 cm to 5 cm in size with central vascularization and was positioned near the left ovary, but it did not incorporate it. The pulsatility index (PI) was 0.69 and the resistance index (RI) was 0.48. Tumor marker levels were not elevated (CEA 1.08 µg/L, CA19-9 < 2 kIU/L, CA125 26.8 kIU/L), and the patient was not experiencing any symptoms.

**Figure 2:**
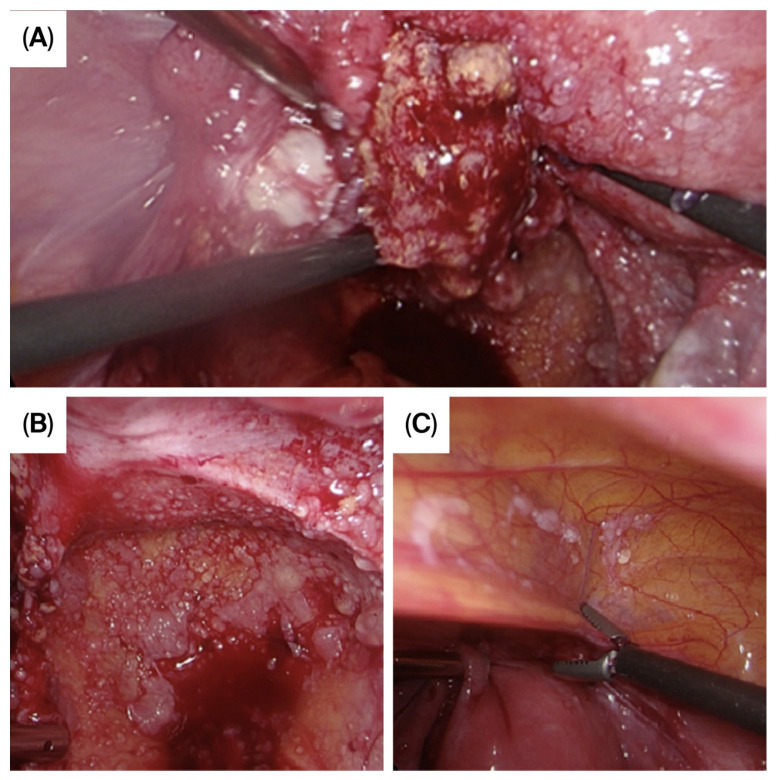
A laparoscopy was performed and a tumor mass with solid appearance was found positioned between left ovary and the uterus (**A**). The tumor mass had a soft consistency and was prone to bleeding during manipulation. During the procedure, another finding was multiple white nodules dispersed on both ovaries, the pelvic peritoneum, the uterus, the vesicouterine fold, the medial umbilical folds, and the abdominal peritoneum (**B**,**C**). Extirpation of the extraovarian tumor mass was performed as well as left adnexectomy. Multiple peritoneal biopsies of white nodules were acquired. Intraoperative pathology consultation determined that the removed tumor was a well-differentiated papillary mesothelial tumor (WDPMT) (**A**). Nodules were less than one cm in size. Histological analysis of peritoneal nodules presented as cystic spaces separated by connective tissue and lined with mesothelium without atypia. They were diagnosed as peritoneal inclusional cysts. Peritoneal inclusional cysts comprise one or more cystic spaces lined by bland mesothelial cells. This is a broad diagnostic category of uncertain pathogenesis, ranging from small (0.1 cm) incidental unilocular cysts to florid (>30 cm) symptomatic multilocular tumors. The WHO advocates for (multiloculated) peritoneal inclusional cyst(s) and discourages (benign) multicystic mesothelioma and (benign) cystic mesothelioma. On microscopic examination of this multiloculated cystic lesion, individual cysts were lined by bland, flattened mesothelial cells. Mitoses were not identified. The cysts were separated by fibrous septa with patchy chronic inflammation. There was no infiltration of underlying tissues. The gross and microscopic findings were most consistent with a diagnosis of multiloculated peritoneal inclusional cyst (previously termed multicystic mesothelioma) [1,2] (**C**). Well differentiated papillary mesothelial tumor (WDPMT) is a neoplastic mesothelial proliferation of low malignant potential, which predominantly involves the peritoneum of young women. Microscopically it is composed of branching plump papillae lined by a single layer of bland cuboidal mesothelial cells. [3] Cytologic atypia is minimal, and mitoses usually absent (or at most 1 mitosis per 10 high power fields). Papillary cores may show myxoid change, edema, psammomatous calcifications, fibrosis, foamy macrophages or chronic inflammation. Invasion of underlying pre-existing tissues is absent. Some cases are associated with a discrete adenomatoid tumor or multicystic mesothelioma with no apparent effect on prognosis. Immunohistochemically these lesions are positive for calretinin (virtually 100%) (cytoplasmic + nuclear staining is most specific), WT1 (virtually 100%), D2-40 (virtually 100%), cytokeratin 5/6 (~90%), PAX8 (60–95% show at least partial staining), Ki67 (<5%), BAP1 (retained in virtually all cases, loss strongly suggests malignant mesothelioma with WDPM-like features) [4,5]. Differential diagnosis includes: 1. Diffuse malignat peritoneal mesothelioma which is characterized by diffuse involvement of the peritoneum, infiltration of underlying pre-existing tissue, at least moderate cytologic atypia in most cases increased mitotic activity (>1 mitosis per 10 high power fields is typical). Solid architecture may be present, and BAP1 is lost in ~70%. 2. Localized malignant peritoneal mesothelioma, which is an exceptionally rare diagnosis, microscopically characterized by complex papillary or solid architecture, identical to that seen in diffuse malignant peritoneal mesothelioma. Cytologic atypia is at least moderate, and mitotic activity is increased (1 mitosis per 10 high power fields is typical). It may contain WDPM-like foci, so thorough sampling is necessary. 3. Serious borderline tumors are composed of columnar cells with mixed morphology, including ciliated cells. They are negative for calretinin and positive for epithelial lineage markers. Well-differentiated papillary mesothelial tumors and peritoneal inclusional cysts are rare clinical entities. These tumors possess low malignant potential; however, long-term surveillance is warranted [6]. They are usually found in women of reproductive age [7]. Hoekstra et al. reported that the median age of patients in their study was 41 years [7]. The patient presented in our case report was 41 years old. Kim et al. reported 12 clinical cases of WDPMT in their institution, with a median age of patients of 64 years. Ten cases of WDPMT in that study were also incidental findings during surgery, as with our case [8]. Sun et al. reported 75 cases of WDPMT during a 17-year period. The median age of patients in their study was 40 years, and the majority of cases were incidental findings during operative procedures performed for other reasons [9]. Moreover, a lesion size greater than two cm has been reported to be associated with histological findings of WDPMT with adenomatoid tumor or multicystic mesothelioma [9], as with our case. There are no definitive treatment guidelines regarding patients who present with WDPMT and multicystic mesothelioma. Resection with long-term follow-up is usually the suggested approach [10]. Treatment strategy depends on patients’ comorbidities, symptoms, the extent of the tumor, and progression of the disease [10]. Lee et al. reported that additional treatment besides cytoreduction should be considered if the patient has abdominal distension, pain or a large volume of ascites, as these symptoms are associated with poor outcome [11]. Supposed risk factors associated with developing WDPMT are smoking or history of smoking, asbestos exposure, previous abdominal surgeries, family history, endometriosis, and trauma [6,8]. Our patient had only one of these factors, and that was previous abdominal surgery (caesarean section). Also, she was asymptomatic during the period before surgery. Given that the clinical course of WDPMT and multicystic mesothelioma is usually indolent and that our patient had no symptoms associated with poor outcome, we opted for the resection of the main tumor mass adherent to the left adnexa and performed multiple biopsies of the peritoneum covered with miliary nodules. Complete debulking due to the extent of the lesions was not possible. In addition, Lee et al. reported that the efficacy of radical surgery beyond simple excision is questionable [11]. Furthermore, it is reported that adjuvant therapy is not without potential side effects and ought to be used cautiously [1,3,4]. Almost three years after the procedure, our patient is still well and without symptoms. Regular clinical and ultrasound examinations were performed every six months. There was no clinical or ultrasound suspicion of tumor recurrence or dissemination.

**Figure 3:**
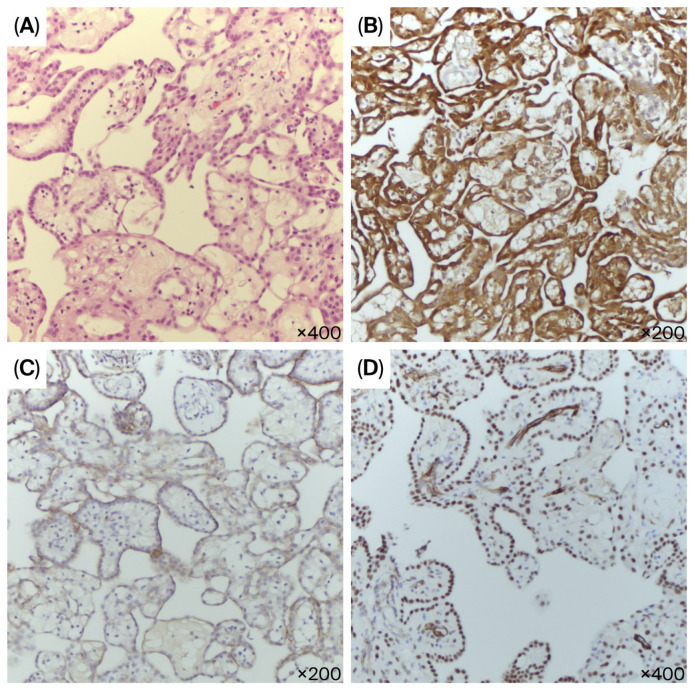
(**A**) H&E stain, (**B**) calretinin-positive cells, (**C**) podoplanin-positive cells and (**D**) WT1-positive cells. Histological findings of the tumor mass presented as edematous papillae lined with cuboid cells whose stroma was partly edematous and partly hyalinized. There was no cell atypia or mitotic activity (**A**). Immunohistochemical analysis showed that cells had positive staining for calretinine (**B**), WT1 (**D**) and podoplanin (**C**) and negative for CK5/6. These results are in concordance with those of other studies [3,4,5]. No stromal invasion was detected. Other studies have also presented similar histological findings of WDPMT [6,7,8,9,11,12].

## Data Availability

The original contributions presented in this study are included in the article. Further inquiries can be directed to the corresponding author.
